# A New Prenylated Indole Diketopiperazine Alkaloid from *Eurotium cristatum*

**DOI:** 10.3390/molecules191117839

**Published:** 2014-11-03

**Authors:** Xianwei Zou, Ying Li, Xiaona Zhang, Qian Li, Xuan Liu, Yun Huang, Tao Tang, Saijing Zheng, Weimiao Wang, Jintian Tang

**Affiliations:** 1 Key Laboratory of Particle & Radiation Imaging, Ministry of Education, Department of Engineering Physics, Tsinghua University, Beijing 100084, China; E-Mails: zouxw000@126.com (X.Z.); liying_90126@126.com (Y.L.); zxn_1228@sina.com (X.Z.); yaoyaoldlq@163.com (Q.L.); liux_54@163.com (X.L.); sungood591@sina.com (Y.H.); tao_tang1@126.com (T.T.); 2 Shanghai Tobacco Group Corporation, Shanghai 200082, China; E-Mails: zhengsj@sh.tobacco.com.cn (S.Z.); zdsys@sh.tobacco.com.cn (W.W.); 3 Beijing Longcredit Essence Biotechnology Co. Ltd, Beijing 100085, China

**Keywords:** *Eurotium cristatum*, diketopiperazine, radical scavenging activity

## Abstract

A new prenylated indole diketopiperazine alkaloid, cristatumin F (**1**), and four known metabolites, echinulin (**2**), dehydroechinulin (**3**), neoechinulin A (**4**) and variecolorin O (**5**), were isolated from the crude extract of the fungus *Eurotium cristatum*. The structure of **1** was elucidated primarily by NMR and MS methods. The absolute configuration of **1** was assigned using Marfey’s method applied to its acid hydrolyzate. Cristatumin F (**1**) showed modest radical scavenging activity against DPPH radicals, and exhibited marginal attenuation of 3T3L1 pre-adipocytes.

## 1. Introduction

**Figure 1:**
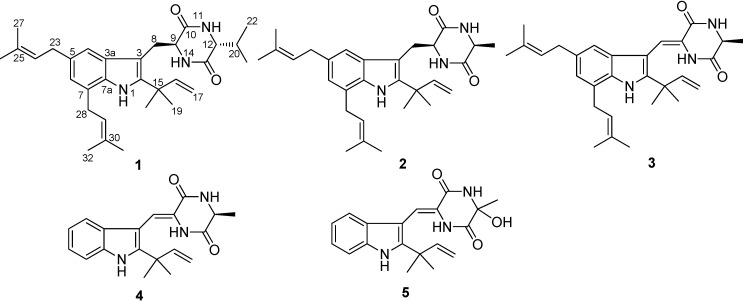
Chemical structures of compounds **1**–**5**.

Prenylated indole diketopiperazines are an important class of compounds with diverse chemical structures and biological activities [1]. Prenylated indole alkaloids, generally characterized by a reverse isoprenic chain in the C-2 position of the indole nucleus, are often found in fungal sources. This group of alkaloids are an important class of molecules possessing a variety of biological activities, including antibacterial [2,3], immunosuppressive [4,5], brine shrimp inhibition [3], radical scavenging [6,7], UV-A protecting [7,8], and cytotoxicity properties [9]. Chinese fuzhuan brick tea is a unique microbially fermented tea characterized by a period of fungal growth, especially of the fungus *Eurotium cristatum*, during its manufacturing process [10]. Biologically active secondary metabolites have been isolated from the same fungus, including diketopiperazines with antibacterial and insecticidal activity [3], eurocristatine, a diketopiperazine dimer from the marine sponge-associated *E. cristatum* KUFC 7356 [11], and benzaldehyde derivatives with *in vitro* anti-cancer antivity against human tumor cell lines [12,13]. During our ongoing search for new bioactive natural products from unique fungal species, a subculture of an isolate of *E. cristatum*, a fungus isolated from a sample of fuzhuan brick tea, was grown in solid-substrate fermentation culture. Fractionation of its organic solvent extract led to the isolation of a new prenylated indole diketopiperazine alkaloid, named cristatumin F (**1**), together with several known metabolites, including echinulin (**2**) [4], dehydroechinulin (**3**) [9], neoechinulin A (**4**) [8] and variecolorin O (**5**) [14] (Figure 1). Details of the isolation, structure elucidation, and biological activities of these compounds are reported herein.

## 2. Results and Discussion

**Figure 2:**
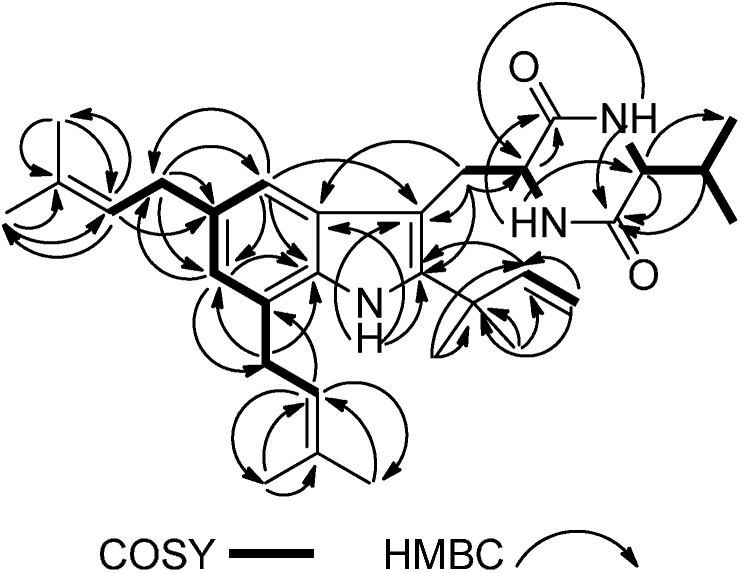
Selected 2D NMR correlations for **1**.

Cristatumin F (**1**) was obtained as a colorless powder with a molecular formula of C_31_H_43_N_3_O_2_ (indicating 12 degrees of unsaturation), determined by HRESIMS. The IR spectrum of **1** showed absorption bands for amine (3459 and 3435 cm^−1^), carbonyls (1676 cm^−1^), and aromatic functionalities (3200 and 1446 cm^−1^) in the molecule. Analysis of the ^1^H, ^13^C (APT experiment), and HSQC NMR spectroscopic data of **1** revealed the presence of eight methyl groups, four methylene units, eight methines, 11 quaternary carbons, 14 aromatic/olefinic carbons (six of which are protonated), and two amide/carboxyl carbons. These data, together with three exchangeable protons accounted for all ^1^H and ^13^C-NMR resonances for **1**, and are consistent with the molecular formula C_31_H_43_N_3_O_2_. Interpretation of the ^1^H–^1^H COSY NMR data of **1** identified four isolated proton spin systems corresponding to the C-4–C-29 (via C-5 and C-7), C-16–C-17, C-8–*N*H-14 (via C-9), and *N*H-11–C-22 (via C-12) fragments. HMBC correlations from CH_3_-26 and CH_3_-27 to C-24 and C-25, H-24 to C-23, C-26, and C-27, CH_3_-31 and CH_3_-32 to C-29 and C-30, as well as correlations H-29 to C-28, C-31, and C-32 established the two prenyl subunits (C-23–C-27 and C-28–C-32). While those from CH_3_-18 and CH_3_-19 to C-15 and C-16, H-17 to C-15 and C-16, and from H-16 to C-18 and C-19 completed the C-15–C-19 substructure of **1**. The tetrasubstituted indole moiety of **1** was deduced by HMBC correlations from H-4 to C-3, C-6, C-7a, and C-23; from H-6 to C-4, C-7a, C-23, and C-28; and from *N*H-1 to C-2, C-3, and C-3a, together with the ^1^H–^1^H COSY correlation between H-4 and H-6. HMBC cross-peaks from H_2_-23 to C-4, C-5, and C-6, from H_2_-28 to C-6 and C-7a indicated that C-23 and C-28 were attached to C-5 and C-7, respectively. HMBC correlations from H-16, CH_3_-18, and CH_3_-19 to C-2 led to the connection of C-2 to C-15. The prenylated indole substructure was established based on above analysis and those relevant ^1^H–^1^H COSY correlations (Figure 2).

Interpretation of the ^1^H, ^13^C, and 2D-NMR spectra, especially HMBC, revealed the peptidic nature of **1**. A diketopiperazine ring was suggested by the characteristic NMR data of two amide carbonyls (C-10, δ_C_ 168.6; C-13, δ_C_ 166.5) and two methines (C-9, δ_H_/δ_C_ 4.42/53.6; C-12, δ_H_/δ_C_ 3.87/60.8). HMBC correlations from H-9 to C-10, *N*H-11 to C-9 and C-13, H-12 to -C-13, and *N*H-14 to C-9, C-10, and C-12 clearly suggested the existence of the diketopiperazine system in **1**. The linkage of the prenylated indole core with the diketopiperazine part through the methylene bridge was revealed by the HMBC correlations from H_2_-8 to C-3 and C-9. Comparison of the ^1^H and ^13^C-NMR spectroscopic data of **1** with those of **2** revealed their structural similarities. The molecular weight of **1** is 28 mass units more than that of **2**, and this number corresponds to the difference in mass between the valine (Val) and the alanine (Ala) residues, suggesting that **1** is a diketopiperazine congener to **2** with an Ala unit replaced by a Val unit in the 2,5-diketopeperazine moiety. Analysis of the ^1^H–^1^H COSY and HMBC data of **1** further confirmed the presence of a Val residue. Key HMBC correlations from *N*H-11, H-12 and H-20 to C-13, from *N*H-14 to C-10 and C-12, and from *N*H-11 to C-9 established the linkage between C-10, C-12 and C-13 (Figure 2). On the basis of these data, the gross structure of cristatumin F was established as shown in **1**.

Compounds **2**–**5** were identified as echinulin (**2**) [4], dehydroechinulin (**3**) [9], neoechinulin A (**4**) [8] and variecolorin O (**5**) [14], respectively, by comparison of the NMR and MS data with reported spectroscopic data.

Marfey’s method [15,16] was applied to determine the absolute configuration of the Val (C-12) residue resulting from acid hydrolysis of cristatumin F (**1**). The 1-fluoro-2,4-dinitrophenyl-5-l-alanine amide (FDAA) derivatives of the acid hydrolyzate of **1** and the authentic d- and l-valines were subjected to HPLC-MS analysis. HPLC analysis of FDAA derivatives of the acid hydrolyzates of **1** gave the same retention time as the sample prepared from authentic d-Val. Therefore, C-12 was assigned the *R* absolute configuration. The relative configuration of **1** could not be directly assigned by selective NOE difference experiments (mixing time of 300 ms) due to the lack of enhancement bewteen H-9 and H-12. However, from biogenetic considerations, C-9 in **1** presumably has the same absolute configuration as in **2**, which was also indirectly supported by the lack of NOE enhancement between H-9 and H-12. In addition, **1** displayed positive optical rotation (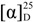 +11.3), while **2** showed negative value (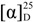 −23.2 (lit. 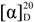 −39.1) [3]), both determined in CHCl_3_. The absolute configuration of **1** was tentatively assigned as 9 *S* and 12 *R* based on the above results. Collectively, these data permitted assignment of structure **1** to the new natural product, cristatumin F. The new compound **1** displayed modest radical scavenging activity against DPPH, with an IC_50_ value of 53.6 μM (ascorbic acid as a positive control, IC_50_ 15 μM), and showed marginal cell prolification inhibition (20.6% inhibition) against 3T3L1 preadipocytes when tested at 200 μM.

## 3. Experimental Section

### 3.1. General

Optical rotations were measured on an Anton Paar MCP 200, and UV data were obtained on a Yoke UV756CRT spectrophotometer. IR data were recorded using a Nicolet Magna-IR 750 spectrophotometer. ^1^H and ^13^C-NMR data were acquired with Bruker Avance-500 spectrometer using solvent signals (CDCl_3_: δ_H_ 7.26/δ_C_ 77.6) as references. The HSQC and HMBC experiments were optimized for 145.0 and 8.0 Hz, respectively. HRESIMS data were obtained using an Agilent Accurate-Mass-Q-TOF LC/MS 6520 instrument equipped with an electrospray ionization (ESI) source. The fragmentor and capillary voltages were kept at 125 and 3500 V, respectively. Nitrogen was supplied as the nebulizing and drying gas. The temperature of the drying gas was set at 300 °C. The flow rate of the drying gas and the pressure of the nebulizer were 10 L/min and 10 psi, respectively. All MS experiments were performed in positive ion mode. Full-scan spectra were acquired over a scan range of *m/z* 100–1000 at 1.03 spectra/s.

### 3.2. Fungal Material

The culture of *E. cristatum* was isolated from a sample of fuzhuan brick tea collected from Yiyang city, Hunan Province, People’s Republic of China, in May 2012. Sequence analysis of the ITS region of the ribosomal DNA suggested highly similarity (98%) with GenBank sequences from the fungus of *E. cristatum* EN220 and *E. cristatum* NRRL 4222. The isolate was identified by one of the authors (T.T.) based on morphology and sequence (GenBank Accession No. KM521200) analysis of the ITS region of the rDNA and assigned the accession number JH-01 in J.T.’s culture collection at Tsinghua Universtiy, Beijing. The fungal strain was cultured on slants of potato dextrose agar (PDA) at 25 °C for 10 days. Agar plugs were cut into small pieces (about 0.5 × 0.5 × 0.5 cm^3^) under aseptic conditions and 15 of these pieces were used to inoculate in three Erlenmeyer flasks (250 mL), each containing 50 mL of media (0.4% glucose, 1% malt extract, and 0.4% yeast extract); the final pH of the media was adjusted to 6.5 and sterilized by autoclave. Three flasks of the inoculated media were incubated at 25 °C on a rotary shaker at 170 rpm for five days to prepare the seed culture. Spore inoculum was prepared by suspension in sterile, distilled H_2_O to give a final spore/cell suspension of 1 × 10^6^/mL. Fermentation was carried out in forty Fernbach flasks (500 mL), each containing 80 g of rice. Distilled H_2_O (120 mL) was added to each flask, and the contents were soaked overnight before autoclaving at 15 psi for 30 min. After cooling to room temperature, each flask was inoculated with 5.0 mL of the spore inoculum and incubated at 25 °C for 40 days.

### 3.3. Extraction and Isolation

The fermented rice substrate was extracted with MeOH (5 × 500 mL), and the organic solvent was evaporated to dryness under reduced pressure to afford a crude extract (230.0 g), which was then suspended in water (700 mL) and successively partitioned with petroleum ether, EtOAc and *n*-BuOH to give solvent dried fractions PE (4.0 g), EA (12.0 g), NBA (9.0 g), respectively.

A portion of PE (4.0 g) was subjected to silica gel VLC using petroleum ether–EtOAc gradient elution. The fraction (84.3 mg) eluted with 40% EtOAc was separated by Sephadex LH-20 column chromatography (CC) eluting with 1:1 CH_2_Cl_2_–MeOH and six fractions 1–6 were collected. Fraction 2 was purified by semipreparative RP HPLC (YMC-Pack ODS-A column; 5 μm; 10 × 250 mm; 85%–98% MeOH in H_2_O for 30 min; 2 mL/min) to afford **1** (3.2 mg, *t*_R_ 20.5 min) and **3** (1.5 mg, *t*_R_ 18.5 min). A portion of EA (12.0 g) fractionated by silica gel VLC using petroleum ether–EtOAc gradient elution. The fraction (1.86 g) eluted with 40%–70% EtOAc was chromatographed on a silica gel (300–400 mesh) column (4.5 × 35 cm) eluting with petroleum ether–Me_2_CO (9:1–1:9). The fraction (67 mg) eluted with 60% Me_2_CO was separated by Sephadex LH-20 CC eluting with MeOH, and then the subfractions were purified by RP HPLC (80%–95% MeOH in H_2_O for 30 min; 2 mL/min) to afford **2** (3.2 mg, *t*_R_ 22.5 min). The fraction (197 mg) eluted with 50% Me_2_CO was separated by Sephadex LH-20 CC eluting with MeOH, and further purification of the resulting subfractions by RP HPLC (55%–70% MeCN in H_2_O for 30 min; 2 mL/min) to afford **4** (5.0 mg, *t*_R_ 27.0 min) and **5** (4.5 mg, *t*_R_ 25.0 min)

### 3.4. Spectral Data

Cristatumin F (**1**): colorless powder; 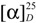 +11.3 (*c* 0.3, CHCl_3_); UV (MeOH) *λ*_max_ (log *ε*) 228 (3.57) nm; IR (neat) ν_max_ 3459, 3435, 3200 (br), 3087, 3058, 2965, 2926, 2874, 1676, 1467, 1446, 1377, 1346, 1102 cm^−1^; ^1^H-, ^13^C-NMR (APT), and HMBC data see Table 1; HRESIMS *m/z* 490.3434 [M + H]^+^ (calcd for C_31_H_44_N_3_O_2_, 490.3428).

**Table 1:** NMR spectroscopic data of cristatumin F (**1**) in CDCl_3_ (δ in ppm).

No.	δ_C_ *^a^*, Mult.	δ_H_ *^b^* (*J* in Hz)	HMBC (H→C)
1-*N*H		8.08, br s	C-2, C-3, C-3a
2	141.5, qC		
3	103.8, qC		
3a	128.9, qC		
4	115.1, CH	7.17, br s	C-3, C-6, C-7a, C-23
5	133.9, qC		
6	122.9, CH	6.83, br s	C-4, C-7a, C-23, C28
7	123.4, qC		
7a	132.2, qC		
8	29.7, CH_2_	3.67, dd (14.7, 3.6)	C-2, C-3, C-3a, C-9
		3.20, dd (14.7, 11.7)	C-2, C-3, C-3a, C-9, C-10
9	53.6, CH	4.42, dd (11.7, 3.6)	C-3, C-8, C-10
10	168.6, qC		
11-*N*H		6.01, br s	C-9, C-13
12	60.8, CH	3.87, br s	C-13, C-21, C-22
13	166.5, qC		
14-*N*H		5.72, br s	C-9, C-10, C-12
15	39.0, qC		
16	145.8, CH	6.11, dd (17.4, 10.6)	C-2, C-15, C-18, C-19
17	112.2, CH_2_	5.19, d (17.4)	C-15, C-16
		5.17, d (10.6)	C-15, C-16
18	27.8, CH_3_	1.53, s	C-2, C-15, C-16, C-19
19	27.7, CH_3_	1.53, s	C-2, C-15, C-16, C-18
20	32.4, CH	2.39, m	C-12, C-13, C-21, C-22
21	18.7, CH_3_	1.05, d (7.0)	C-12, C-20, C-22
22	16.1, CH_3_	0.94, d (6.8)	C-12, C-20, C-21
23	34.6, CH_2_	3.41, d (7.2)	C-4, C-5, C-6, C-25
24	124.5, CH	5.37, t (7.2, 7.2)	C-23, C-26, C-27
25	131.6, qC		
26	17.9, CH_3_	1.83, s	C-24, C-25, C-27
27	25.8, CH_3_	1.76, s	C-24, C-25, C-26
28	31.4, CH_2_	3.55, d (7.0)	C-6, C-7a, C-30
29	122.9, CH	5.45, t (7.0,7.0)	C-28, C-31, C-32
30	132.9, qC		
31	17.9, CH_3_	1.89, s	C-29, C-30, C-32
32	25.7, CH_3_	1.76, s	C-29, C-30, C-31

*^a^*: Recorded at 125 MHz; *^b^*: Recorded at 500 MHz.

### 3.5. Determination of the Absolute Configuration of Val in **1**

A solution of compound **1** (0.5 mg) in 6 N HCl (1 mL) was heated at 105 °C for 24 h. Upon removal of excess HCl under vacuum, the hydrolyzate was placed in a 1 mL reaction vial and treated with 1% solution of 1-fluoro-2,4-dinitrophenyl-5-l-alanine amide (FDAA, 100 μL) in acetone followed by 1.0 M NaHCO_3_ (40 μL). The reaction mixture was heated at 45 °C for 1.5 h and then cooled to RT. The mixture was acidified with 2.0 N HCl (20 μL). Likewise, standard d- and l-valine were derivatized separately. The derivatized hydrolyzate and standard amino acids were subjected to reversed-phase HPLC analysis (DIONEX Acclaim ^®^120 C_18_ column; 5 μm; 4.6 mm × 250 mm; 1 mL/min) at 35 °C using the following gradient program: solvent A, H_2_O (0.1% CH_3_COOH); solvent B, MeCN; linear gradient, 15%–45% of B in A over 45 min with UV detection at 340 nm. The retention times for FDAA derivatives of standard l-valine, cristatumin F hydrolysate, and standard d-valine were 33.49, 41.60, and 41.51 min, respectively.

### 3.6. DPPH Radical Scavenging Assay

DPPH assays were carried out according to the method previously described [17], with slight modifications. In a 96-well microplate, sample at different concentrations in absolute EtOH (50 μL) was added into wells containing 0.02 mM 1,1-diphenyl-2-picrylhydrazyl (DPPH) in EtOH (150 μL) and mixed well. Reaction mixtures was incubated at 37 °C for 30 min. Absorbance was measured at 510 nm by a microplate reader, and percent inhibition was calculated. IC_50_ values express the concentration of sample required to scavenge 50% of the DPPH free radicals. Ascorbic acid was used as a positive control.

### 3.7. Cell Viability Assay

The effect of cristatumin F (1) on cellular viability was assessed by using the MTT assay [18,19]. 3T3L1 cells were seeded at a density of 2000 cells per well in 96-well microtiter plates (Costar, Cambridge, MA, USA) and allowed to adhere. Cells were treated with different concentrations of nordihydrocapsaicin or compound **1** for 24, 48, or 72 h. At each time point, the wells were washed three times with warm phosphate buffer solution (PBS) and incubated again for another 4 h with DMEM containing 5 mg/mL of MTT. After removing the culture medium, 150 μL DMSO was added to dissolve the precipitates and the resulting solution was measured for absorbance at 490 nm using an ELISA reader (iMark, Bio Rad 680, Hercules, CA, USA).

## 4. Conclusions

Cristatumin F (**1**) is a new member of prenylated indole diketopiperazine-type of metabolites, and the presence of a Val unit in **1** is unprecedented. In this work, the discovery of the new indole diketopiperazine alkaloid from *E. cristatum* further expanded the structural diversity of the secondary metabolites produced by this fungal species.
